# Enteroaggregative *Escherichia coli* Have Evolved Independently as Distinct Complexes within the *E. coli* Population with Varying Ability to Cause Disease

**DOI:** 10.1371/journal.pone.0112967

**Published:** 2014-11-21

**Authors:** Marie Anne Chattaway, Claire Jenkins, Dunstan Rajendram, Alejandro Cravioto, Kaisar Ali Talukder, Tim Dallman, Anthony Underwood, Steve Platt, Iruka N. Okeke, John Wain

**Affiliations:** 1 Gastrointestinal Bacteria Reference Unit, Public Health England, London, United Kingdom; 2 Genomic Service Unit, Public Health England, London, United Kingdom; 3 International Vaccine Institute, Gwanak-gu, Seoul, Republic of Korea; 4 Centre for Food and Water Borne Diseases, International Centre for Diarrhoeal Disease Research, Dhaka, Bangladesh; 5 Bioinformatics, PHE, London, United Kingdom; 6 Haverford College, Haverford, Pennsylvania, United States of America; 7 Norwich Medical School, University of East Anglia, Norwich, United Kingdom; Beijing Institute of Microbiology and Epidemiology, China

## Abstract

Enteroaggregative *E. coli* (EAEC) is an established diarrhoeagenic pathotype. The association with virulence gene content and ability to cause disease has been studied but little is known about the population structure of EAEC and how this pathotype evolved. Analysis by Multi Locus Sequence Typing of 564 EAEC isolates from cases and controls in Bangladesh, Nigeria and the UK spanning the past 29 years, revealed multiple successful lineages of EAEC. The population structure of EAEC indicates some clusters are statistically associated with disease or carriage, further highlighting the heterogeneous nature of this group of organisms. Different clusters have evolved independently as a result of both mutational and recombination events; the EAEC phenotype is distributed throughout the population of *E. coli*.

## Introduction

The definition of EAEC varies in studies which either use its aggregative adherence (AA) phenotype on HEp-2 cells [1], the CVD432 probe [2] or PCR to detect the anti-aggregative transporter (*aat*) gene [3] or the EAEC regulatory gene (*aggR*) [4] or a combination of phenotype and genotype. Enteroaggregative *E. coli* (EAEC) have been associated with diarrhoea in epidemiological studies and outbreaks. Investigations of EAEC are based on identification of a group of bacteria (EAEC) assumed to be pathogenic as they were isolated from symptomatic cases. However, not all *E. coli* which contain EAEC virulence factors are pathogenic [5], [6] and so associations between EAEC and virulence are not clear. A comprehensive study looking at the relationship between phylogeny from case or healthy carriage in multiple countries has not been performed and there has been limited analysis of EAEC at the population level. The most detailed study on EAEC population analysis was in Nigeria and was carried out to find an association with EAEC complexes and disease in children under 5 with links to virulence genes, resistance and plasmid groups [7]. Results indicated that the range of sequence types (STs) associated with EAEC is very large and disease, only within a specific age-group, was linked to ST10, an ST associated with multiple *E. coli* pathotypes. There were no reported associations between disease and, virulence genes, resistance profiles, nor plasmid compatibility groups.

Serogrouping (typing of the somatic antigen only) and serotyping (typing of the somatic and flagella antigen) is used extensively for characterising and classifying *E. coli* and *Salmonella enterica*. For both species serogroup is not discriminatory enough to be a useful strain typing tool but serotype can be more robust. For *Salmonella*, serotype is strongly associated with sequence type [8]. Serotyping therefore can give a robust typing scheme although conversion between serotypes can occur by horizontal genetic exchange [8] and so distort the relationship within serotypes. The relationship between serotype and the EAEC phenotype is not defined; here we describe a comprehensive examination of the relationship between phylogeny/serotype/sequence type and whether the strain was isolated from a patient with diarrhoea (case) or a healthy control.

We addressed the questions, are certain EAEC lineages more likely to be associated with disease and have all EAEC evolved from a common ancestor? The study used globally sourced EAEC isolates from three major case control studies and analysed chromosomal core sequence data to look for an association between bacterial background and disease.

## Materials and Methods

### Bacterial Strains

Three case control studies, sporadic and outbreak cases of 564 EAEC spanning over 29 years (1985–2013) were used in this study (Table 1). All of these strains were included to encompass a representation of EAEC in the global community (including UK travellers) over the past three decades. EAEC were defined as having the *aat* gene/CVD432 probe reaction [2], [3], and/or the *aggR* regulatory gene [6] and/or the aggregative adherence (AA) phenotype [1] where the phenotypic test was available (Table 1). Isolates included strains from multiple studies including the UK (273), Bangladesh (169), Nigeria (121) and the prototypical 042 EAEC reference strain from Peru (1) (Table 1). Due to the varying definition of EAEC, all strains were included irrespective of phenotypic and genotypic definition to prevent any bias that may affect the analysis. Where an EAEC outbreak was related to one ST and serotype, only one representative strain has been included.

**Table 1:** Summary of 564 EAEC strains analysed in this study.

Country	Source	Year Range	Case	Control	Reference
Peru	∞042 prototypical strain	1985	1	0	[27]
UK	#GBRU Archive Clinical strains	1985–1995	17	0	This Study∞
UK	∞IID1 Case/Control Study	1993–1996	121	36	[28]
UK	∞GBRU Outbreak A	1994	2	0	[29]
UK	∞GBRU Outbreak B	1994	8	0	[29]
UK	∞GBRU Outbreak C	1994	1	0	[29]
UK	∞GBRU Outbreak D	1995	3	0	[29]
Bangladesh	∞GBRU Outbreak E	1998	12	0	This Study∞
Nigeria	∞Nigeria Case/Control Study	1999	66	55	[7]
UK	#IID2 case study	2008–2009	25	0	[5]
Bangladesh	∞GEMS Case/Control Study	2007–2011	97	61	[30], [31]
Germany	#O104:H4 VTEC Outbreak	2011	1	0	[26]
UK	#O111:H2 Household Outbreak	2012	1	0	[32]
UK	#GBRU Clinical Strains	2009–2013	38	0	This Study∞
UK	#GBRU Spice Outbreak	2013	19	0	[33]

Selection of EAEC strains used in this study including the year the strain was isolated and its geographical location. ∞Strains from this study not previously described include archived clinical strains received by GBRU for typing between 1985–1995, Outbreak E of enteroaggregative *E. coli* that occurred in Bangladesh in 1998, recent clinical strains received by GBRU for typing between 2009–2013. #EAEC were defined as having the *aat* and/or *aggR* gene. ∞Other EAEC strains were defined as having the *aat* gene/CVD432 probe reaction and/or the aggregative adherence (AA) phenotype.

Nigeria isolates were previously analysed [7] All other EAEC strains were plated onto blood agar plates (PHE Media) to test for purity and archived onto Dorset Eggs (PHE Media) and stored at room temperature and also archived on beads [Prolab] and stored at −80°C.

### Identification and Serotyping

Identification of UK and Bangladesh enteroaggregative *Escherichia coli* (EAEC) strains (443 strains) was confirmed phenotypically using biochemical profiling of media tubes [9] by the Gastrointestinal Bacteria Reference Unit of PHE at Colindlae. Typical metabolic profiles of *E. coli* included positive reactions for glucose, gas, lactose, mannitol, lysine, ornithine, mucate, sodium acetate and indole. Serotyping of the somatic and flagella antigen [10] was carried out on the heat stable lipopolysaccharide (Somatic or O) antigens and the flagellar (H) antigens. Strains which reacted with all antigens were termed rough and those that did not react with any were termed ‘O?’ or ‘H?’. Nigerian strains had previously been identified and published [7], strains were not accessible for serotyping.

### Multi-locus sequence typing and analysis of EAEC

Nigerian sequence data was provided by Okeke *et al* as previously published [7]. Genomic DNA Extraction of all other *E. coli* isolates was carried out using the Wizard Genomic DNA purification kit (Promega). PCR amplification of seven Multilocus sequence typing (MLST) gene targets; *adk*, *fum*C, *gyr*B, *icd, mdh*, *pur*A and *rec*A [11] was carried out followed by PCR purification of the amplicons using the ExoSAP-IT PCR cleanup method (Amersham Biosciences UK Ltd). Purified PCR fragments from the seven MLST gene targets were sequenced with both forward and reverse sequencing primers using the ABI prism Bigdye Terminator v3.1 Cycle Sequencing kit (Applied Biosystems) and detected and analysed on the 3730XL ABI Genetic Analyser (Applied Biosystems). Sequence data was analysed and checked for quality and alleles trimmed for analysis, any ambiguous results were repeated (BioNumerics v6.1). Allele numbers and sequence types (ST) were calculated and deposited in the publically accessible *E. coli* MLST database (http://mlst.warwick.ac.uk.). Phylogenetic inference of the EAEC complexes ancestral allelic profiles and strain interrelatedness were made using minimum spanning trees (BioNumerics v6.1). A complex (Cplx) included any single locus variants (SLV) of an allele in relation to a ST.

### Selection of EAEC Disease and Carriage complexes and statistical analysis

As of 18^th^ December 2013, the data available in the public database indicates there were 155 EAEC (121 *Okeke et al* Nigerian study used in this study excluded) out of 6110 *E. coli* entries, accounting for 2.4% of the database. There were 1164 entries of defined diarrhoeagenic pathotypes (see below for description) of *E. coli* which EAEC accounts for 13 % (155/1164). From the 564 strains used in this study, a complex was considered a successful representation if it contained 4 or more strains which would account for a minimum of 2.5% (4/155) of the known EAEC deposited in the public database. The majority of the MLST data associated with these isolates has been previously published [7], [11].

From the EAEC dataset used in this study, complexes containing four or more EAEC were deemed successful (i.e. strains which have continued to proliferate over time in the population) of which there were 17 complexes. The 17 assigned complexes were then tested using a fishers exact test [12] for the significance of the complexes being associated with disease or carriage in relation to the entire dataset (564 strains). Statistical tests of significance were conducted using the Fisher's exact test on Epi-Info version 2.3.1 (http://www.openepi.com).

The public database was compared against each of the 17 complexes to rule out complexes with a high association with other pathotypes [11]. Pathotypes included diarrheagenic types including enterotoxigenic, verocytotoxic, enteropathogenic, enteroinvasive and diffusely adherent *E. coli* (ETEC, VTEC, EPEC, EIEC and DAEC respectively). Extra-intestinal pathogenic *E. coli* (ExPEC) including wounds, meningitis, external sources (ExPEC_Vag) and urinary pathogenic *E. coli* (UPEC). Antibiotic resistance *E. coli* (ESBL, CTX-M-15, NMEC, AmpC CYM-2, c CMY-2, NDM-1, ESBL CTX-M-32 & OXA-48). Other pathotypes included avian pathogenic *E. coli* (APEC), non-pathogenic commensal strains and *E. coli* with no defined pathotype. EAEC complexes were assessed based on the public database and data from this study and tested using a fisher exact test [12] (open epi version 2.3.1) for significance of the complexes being associated with EAEC.

### ClonalFrame Analysis

Clonal Frame analysis was carried out (http://www.xavierdidelot.xtreemhost.com/clonalframe.htm) on all EAEC isolates to investigate the relationships of the different sequence type complexes. ClonalFrame is a Bayesian method of constructing evolutionary histories that takes both mutation and recombination into account [13]. The Graphic User Interface in the ClonalFrame programme was used to construct 75% majority-rule consensus trees, mutational (theta) and recombination rates. Other analysis including the measure of the frequency at which recombination occurs relative to mutation (ρ/θ). The relative effect of recombination on the genetic diversification of populations, ratio r/m in which the ratio of rates at which nucleotides become substituted as a result of recombination and mutation [14] was also used. Finally, the external to internal branch length ratio was computed which gave the inferred expected values against the coalescent and actual ratios. Analysis was split into assessing the Bangladesh and Nigeria case control studies and UK clinical data set for comparison against the entire dataset.

### Placing EAEC in the *E. coli* phylogeny

Multi-locus sequence analysis (MLSA) was performed by concatenating MLST sequence alleles of the EAEC from this dataset and all sequence types representative of the *E. coli* phylogeny. These were aligned and clustered (MEGA V 5.1) and the genetic relationship of isolates designated as was assessed in the context of all *E. coli* using a neighbour joining tree phylogeny (MEGA V 5.1 and FigTree V 1.4). Phylogrouping PCR was carried out on the 17 main groups of EAEC [15] and labelled on the phylogeny.

## Results

### Serotype and complex distribution within the EAEC population structure

From the 564 EAEC strains studied, there were 126 different sequence types, including additional not previously described sequence types of which 57 were single locus variants (SLV), 20 double locus variants (DLV) and two were triple locus variants (TLV).

**Figure 1:**
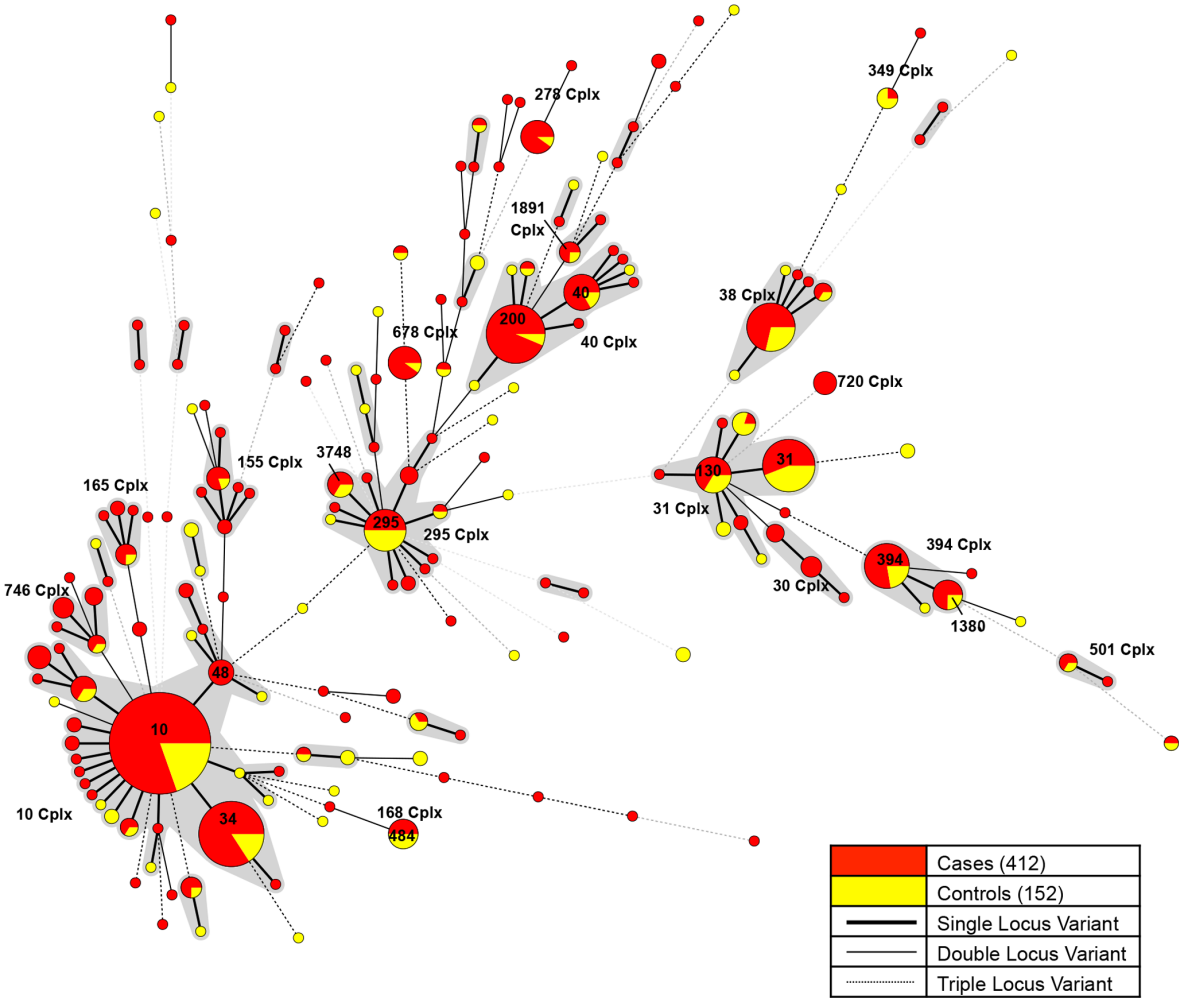
Minimal spanning tree of 564 enteroaggregative *E. coli.* Minimum spanning tree of the 564 EAEC used in this study colour coded by isolates from cases (red) and controls (yellow). Complexes shaded in grey consist of single locus variants (SLV). Sequence types and complex (Cplx) are labelled as numbers.

There were 17 main complexes (Figure 1) containing 4 or more strains of EAEC totalling 358 strains with the top five complexes (Cplx) including ST10 Cplx (39%, 141/358), ST31 Cplx and ST40 Cplx (12%, 42/358), ST394 Cplx (7%, 26/358) and ST295 Cplx and ST38 Cplx (6%,21/358). There were 35 isolates (6.2%, 35/564) that contained one or more new alleles (40 new alleles in total) not previously described. All new alleles were deposited to the public database (http://mlst.ucc.ie/mlst/dbs/Ecoli) for a new allele and/or ST assignment.

Most EAEC serotypes were heterogeneous with respect to ST and dispersed throughout the population structure (Figure S1): Some serotypes were predominantly associated with STs (O7:H4-ST484, O104:H4-ST678, O111:H21-ST40, O125ac:H9-ST295, O153:H30-ST38,) while others were found in multiple STs (O44:H18-ST449, ST414, ST30, O126:H27-ST200 & SLV, ST155, O166:H15-ST349 & SLV/DLV, ST130, ST394,). There were no mutually exclusive ST and serotypes found in the EAEC population structure (Table S1).

### EAEC complexes associated with disease and carriage

The population structure of EAEC was heterogeneous containing 17 complexes (either single ST or complexes) of successful lineages containing 4 or more EAEC (Figure 1, Table 2).

**Table 2:** Assessment of EAEC complexes associated with cases or controls.

Group	ST complex	UK	Nigeria	Bangladesh	Case	Control	Total	Total % of EAEC	CASE: CONTROL %	P Value
Group 1	10	128	24	21	138	35	173	30.7	80∶20	0.01
Group 2	40	39	1	12	44	8	52	9.2	85∶15	0.03
Group 3	31	27	11	12	28	22	50	8.9	56∶44	0.005
Group 4	295	13	2	21	24	12	36	6.4	67∶33	0.24
Group 5	38	3	4	21	19	9	28	5.0	68∶32	0.33
Group 6	394	9	10	8	20	7	27	4.8	74∶26	0.56
Group 7	746	9	1	1	10	1	11	2.0	90∶10	0.16
Group 8	155	0	1	9	9	1	10	1.8	90∶10	0.2
Group 9	678	8	0	2	9	1	10	1.8	90∶10	0.2
Group 10	278	7	1	2	9	1	10	1.8	90∶10	0.2
Group 11	168 (ST484)	0	4	5	5	4	9	1.6	56∶44	0.2
Group 12	30	7	0	0	8	0	8	1.4	100∶0	0.08
Group 13	165	3	0	5	7	1	8	1.4	83∶17	0.32
Group 14	1891	0	0	5	4	1	5	0.9	80∶20	0.59
Group 15	720	0	0	5	5	0	5	0.9	100∶0	0.21
Group 16	501	2	2	0	3	1	4	0.7	75∶25	0.71
Group 17	349	0	1	3	1	3	4	0.7	25∶75	0.06
Totals	-	248	62	132	343	107	442	-	-	-
Whole Data Set	-	273	121	169	412	152	564	-	-	-

Assessment of the successful EAEC complexes (>4 strains) as to the association with cases or controls and showing the data of EAEC numbers according to complex size, Country and association with case or control. Groups are in order of complex size from the largest to smallest. Probability (Fishers exact test) of the group being significantly associated with case or control is tabulated at the end.

There was a 2.7∶1 ratio of case isolates to controls in this study. Complexes with a higher ratio in cases were deemed associated with cases and complexes with a higher ratio in controls were deemed associated with controls, complexes that were below this ratio were deemed to be not associated with cases or controls. This resulted in eleven complexes being associated with disease (ST10, 30, 40, 155, 165, 278, 501, 678, 720, 746 and 1891, Cplx), two complexes associated with carriage (ST31 and 349 Cplx) and four complexes neither associated with disease or carriage (ST,38, 168, 295 and 394 Cplx).

The disease complexes and carriage complexes were combined and statistical analysis showed both of the disease and carriage complexes were statistically significant (P = <0.001 and P = 0.001 respectively) (Table 2).

Individual complexes were then tested for statistical association with disease or carriage which showed ST10 Cplx and ST40 Cplx were independently statistically significantly (P = 0.01 & 0.03 respectively) associated with disease. ST31 was independently statistically significantly (Fishers chi-square, p = 0.005) associated with carriage (due to the fact that there was a higher ratio of controls).

Situating the 17 successful EAEC complexes identified in this study within the global *E. coli* phylogeny as represented in the public database (Table 3) showed that with the exception of ST155 Cplx, all complexes were significantly associated with being EAEC pathotype (P≤0.01).

**Table 3:** Assessment of EAEC associated with other pathotypes.

Group	ST complex	EAEC (This study)	EAEC Public	EPEC	ETEC	STEC	EIEC	DAEC	Commensal	No Pathotype	Other pathotypes	Total DEC	Total *E.coli* inc. EAEC	Other *E.coli* total	%EAEC: DEC	%EAEC: *E.coli*	Total EAEC	P value
Group 1	10	149	42	17	22	4	0	0	5	141	83	234	463	272	81.6	41.3	191	<0.001
Group 2	40	51	8	4	0	3	0	0	0	2	0	66	68	9	89.4	86.8	59	<0.001
Group 3	31	39	19	0	0	0	0	0	0	6	8	58	72	14	100.0	80.6	58	<0.001
Group 4	295	34	1	3	0	0	0	0	1	0	2	38	41	6	92.1	85.4	35	<0.001
Group 5	38	24	4	0	0	1	0	0	0	10	27	29	66	38	96.6	42.4	28	<0.001
Group 6	394	17	11	0	0	0	0	0	0	3	2	28	33	5	100.0	84.8	28	<0.001
Group 7	746	10	0	0	4	0	0	0	0	0	1	14	15	5	71.4	66.7	10	<0.001
Group 8	155	9	2	1	3	1	1	0	2	27	22	17	68	57	64.7	16.2	11	0.11
Group 9	678	10	0	0	0	0	0	0	0	1	0	10	11	1	100.0	90.9	10	<0.001
Group 10	278	9	0	0	0	0	1	0	0	0	0	10	10	1	90.0	90.0	9	<0.001
Group 11	168 (ST484)	5	4	0	0	0	1	2	0	10	8	12	30	21	75.0	30.0	9	0.003
Group 12	30	8	2	1	0	0	0	0	0	0	0	11	11	1	90.9	90.9	10	<0.001
Group 13	165	8	0	1	7	3	0	0	0	6	1	19	26	18	42.1	30.8	8	0.005
Group 14	1891	5	0	0	0	1	0	0	0	0	0	6	6	1	83.3	83.3	5	<0.001
Group 15	720	5	0	0	0	1	0	0	0	0	1	6	7	2	83.3	71.4	5	<0.001
Group 16	501	2	1	0	0	0	0	0	0	0	0	3	3	0	100.0	100.0	3	<0.001
Group 17	349	3	1	2	0	0	0	0	1	0	2	6	9	5	66.7	44.4	4	0.01

Assessment of the successful EAEC complexes (>4 strains), as to the association with the complexes being associated with EAEC or other *E. coli* pathotypes in the public database (all data from 18.12.2013) including commensal, diarrhoeagenic and extra-intestinal *E. coli*. Nigerian dataset is included under the public database, UK and Bangladesh dataset is included under EAEC (This study). See methods for description of pathotypes included. Total EAEC included is 598 strains (443 from this study plus 155 EAEC from public database strains), other *E. coli* total is 6076 strains (6674 minus 598 EAEC and minus 141 *Shigella* isolates included in the public database). Probability (Fishers exact test) of the group being significantly associated with EAEC or other pathotypes is tabulated at the end.

### Evolutionary Events leading to successful EAEC disease complexes

ClonalFrame analysis showed that EAEC mutation and recombination rates varied across the complexes and Countries (Table 4 & 5). Complex ST10 Cplx had the highest mutation rate (4.05) and recombination rate (1.2) whereas ST295 Cplx the lowest mutation rate (0.02) and lowest recombination rate (0.002). However, both of these complexes had a similar mutation to recombination ratio. Recombination had the greatest impact (on the diversification of the lineages) on ST40 Cplx (12) and ST394 Cplx (10). Recombination occurred 1.7 times more often than mutation rate among isolates from Bangladesh and Nigeria whereas among strains isolated in the UK, recombination and mutation rate was almost equal. The entire dataset recombination events occurred 1.3 times more often than mutational events.

**Table 4:** Mutation and Recombination rates of dataset by geographical source and all Sequence types found in dataset.

Parameters	Bangladesh N = 169 (108 Cases, 61 Controls)	Nigeria N = 121 (66 cases, 55 controls)	UK N = 254 (228 cases, 36 controls)	All ST N = 199 (138 cases, 61 controls)
**Mutation Rate (theta 0)** Mutational rate & assumed to be constant on the branches of topology	mean: 15.03, credibility_region: 6.95–26.14	mean: 120.79, credibility_region: 69.29–33.00	mean: 70.13, credibility_region: 49.35–94.01	mean: 16.01, credibility_region: 8.64–23.71
**Recombination rate (R)** recombination rate & assumed constant on branches of topology	mean: 22.58, credibility_region: 14.05–33.46	mean: 31.38, credibility_region: 19.68–43.37	mean: 15.66, credibility_region: 9.84–22.31	mean: 89.53, credibility_region 64.21–121.96
**view rho over theta (p/0)** How often recombination occurs relative to mutations	mean: 1.65, credibility_region: 0.77–3.14	mean: 1.68, credibility_region: 0.78–3.80	mean: 1.048907, credibility_region: 0.50–1.987	mean: 1.317856, credibility_region: 0.76–2.07
**view r over m (r/m)** The impact of how important the effect of recombination was in the diversification of the sample relative to mutation	mean: 4.38, credibility_region: 2.38–8.05	mean: 4.10, credibility_region: 2.13–8.09	mean: 2.60, credibility_region: 1.44–4.39	mean: 2.87, credibility_region 1.94–4.24
**External to Internal Branch Length Ratio** Gives the inferred expected values against the coalescent and actual rations. It they are significantly apart then it shows there was a genetic event such as recombination that led to these values.	mean: 0.73, interval: 0.54–0.94 **Significance: 0.00**	mean: 0.56, interval: 0.40–0.76 **Significance: 0.01**	mean: 0.67, interval: 0.50–0.88 **Significance: 0.00**	mean: 0.90, interval: 0.72–1.06 **Significance: 0.00**

ClonalFrame mutation and recombination rates shown as well the impact of recombination over mutation in the diversification of the data and also the significance of the expected value over the inferred value as to whether the data evolved over a period of time (not significant) or due to a large genetic event (significant). This analysis was applied to the different geographical locations, and all 564 EAEC ST found in this study.

**Table 5:** Mutation and Recombination rates of dataset by ST complex.

Parameters	ST10 Cplx & DLV	ST38 Cplx & DLV	ST40 Cplx & DLV	ST295Cplx & DLV	ST394Cplx & DLV	ST31 & ST 130Cplx & DLV
**Mutation Rate (theta 0)**	mean: 4.04, credibility_region: 2.097–6.31	mean: 0.28, credibility_region: 0.02–1.00	mean: 0.94, credibility_region: 0.02–2.62	mean: 0.02, credibility_region: 0.00–1.87	mean: 0.23, credibility_region: 0.00–1.00	mean: 0.65, credibility_region:0.13–1.48
**Recombination rate (R)**	mean: 1.24, credibility_region: 0.41–2.84	mean: 0.08, credibility_region: 0.00–0.38	mean: 0.61, credibility_region: 0.00–1.90	mean: 0.00, credibility_region: 0.00–0.01	mean: 0.10, credibility_region: 0.00–0.46	mean: 0.37, credibility_region: 0.03–0.97
**view rho over theta (p/0)**	mean: 0.33, credibility_region: 0.09–0.82	mean: 0.68, credibility_region: 0.00–3.60	mean: 5.55, credibility_region: 0.00–46.86	mean: 0.57, credibility_region: 0.00–4.49	mean: 4.07, credibility_region: 0.00–33.12	mean: 1.07, credibility_region: 0.04–5.63
**view r over m (r/m)**	mean: 1.20, credibility_region: 0.39–2.66	mean: 3.55, credibility_region: 0.01–19.63	mean: 12.00, credibility_region: 0.00–102.35	mean: 0.91, credibility_region: 0.00–7.04	mean: 10.39, credibility_region: 0.00–74.56	mean: 4.27, credibility_region: 0.24–20.06
**External to Internal Branch Length Ratio**	mean: 0.48, interval:0.28–0.72 **Significance:0.02**	mean: 0.77, interval:0.30–1.51 **Significance:0.15**	mean: 0.64, interval:0.30–1.20 **Significance:0.09**	mean: 0.64, interval:0.29–1.25 **Significance:0.15**	mean: 0.64, interval:0.23–1.32 **Significance:0.24**	mean: 0.56, interval:0.27–1.143 **Significance:0.12**

ClonalFrame mutation and recombination rates shown as well the impact of recombination over mutation in the diversification of the data and also the significance of the expected value over the inferred value as to whether the data evolved over a period of time (not significant) or due to a large genetic event (significant). This analysis was applied to the large main complexes including single locus variants (SLV) and double locus variants (DLV).

The geographical location of the place of isolation of an EAEC strain bears no significance in its phylogeny grouping (with the exception of small geographical specific STs possibly due to sampling bias) and successful EAEC ST were distributed globally (Figure S2) The impact of recombination in the diversification of the sample set relative to mutation showed the greatest impact in the Bangladesh strain set, and the least impact in the strains from the UK. This data suggest that recombination may play an important role in the evolution of EAEC (Table 4 & 5).

External to Internal Branch Length Ratio gave coalescent expectations indicating that all EAEC irrespective of location and including the entire dataset were significantly different (p = <0.001) from the inferred value (Table 4).

### Evolution of EAEC in the context of the *E. coli* population

**Figure 2:**
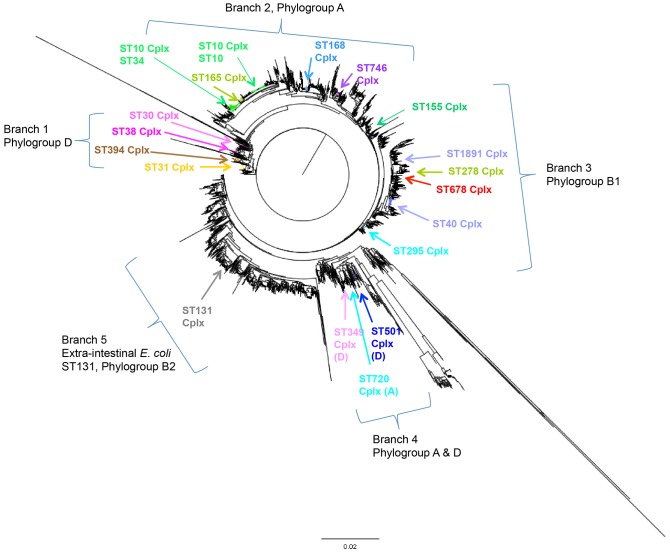
Neighbour joining tree of all *E. coli* and enteroaggregative *E. coli* in this study. Neighbour joining tree of concatenated MLVA of the 564 EAEC used in this and all ST across the *E.* coli population structure. Phylogeny is separated into four main branches. EAEC is distributed throughout the *E. coli* phylogeny as shown in branches 1–4 containing phylogroups, A, B1 and D. The main EAEC complexes was not found in branch 5, phylogroup B2 associated with extra-intestinal infections.

Of the five main branches of *E. coli* phylogeny, EAEC are most prominent on branches 1, 2 and 3 (Figure 2) consisting of phylogroups D, A and B1 respectively. ST30, 31, 38, and 394 Cplxs which are grouped together by MLST population structure (Figure 1) are all located on branch 1 of the *E. coli* phylogeny. The other large successful complexes are dispersed throughout branch 2 and 3. ST10 Cplx shows that some SLVs on the MLST structure are separate in the context of the *E. coli* phylogeny though still closely related. ST295 Cplx which is linked to ST10 Cplx by ST48 is on the opposite end of branch 2 and therefore evolutionary distant. The smaller successful complexes with only 4 EAEC were found at the end of branch 4 which contained a mixture of phylogroups A and D. None of the main EAEC complexes (Table 2) were found in branch 5 of the *E coli* phylogeny which is generally associated with extra-intestinal infections such as ST131 belonging to phylogroup B2.

## Discussion

### Serotyping does not always correlate with genetic relatedness and cannot be used to infer genetic background

Although there were serotypes exclusively from cases (O3:H2, O44:H18, O104:H4, O111:H21, O126:H27and O134:H27), In this study we found no link between a sequence type and a single serotype Although some serotypes were associated with single clonal complexes, they were not mutually exclusive and high recombination rates in some lineages meant that a given serotype could also be distributed in different complexes (Figure S1, Table S1).

Since the development of sequence based typing, such as MLST, the use of traditional typing methods, such as serotyping as a means of population structure [16], [17] have come under close scrutiny. Other studies have also shown that the same serogroups are found in genetically unrelated strains of *E. coli* indicating possible horizontal gene transfer [18] of the cassette encoding the serogroup genes. In this study we were looking for lineages of EAEC and so we used MLST as the primary typing method. and we conclude, as others have, that serotyping is not a suitable method for determining ancestral relatedness of EAEC.

### There are successful multiple lineages of EAEC complexes that are globally distributed

We have shown a statistically significant association of certain sequence type complexes of enteroaggregative *E. coli* with disease or carriage. These complexes represent independent lineages which were spread throughout the entire *E. coli* population (Figure 2) and included the EAEC published complexes in the public database: ST10 Cplx, ST40 Cplx, ST38 Cplx, ST394 Cplx and ST349 Cplx [7]. Prototypical EAEC strains 042 (from Peru) and 17–2 (from Chile) belong to ST31 Cplx and ST10 Cplx respectively, which were prominent in this study. This study also identified MLST complexes that were not currently represented in the public database as associated with the aggregative phenotype including 

ST130 Cplx, ST295 Cplx, ST484 Cplx, ST678 and ST720 Cplx. This data represents a snapshot of EAEC, from three different countries, and the addition of strains across the globe will expand the number STs associated with EAEC. It should be noted that the public database is biased towards *E. coli* of clinical interest such as pathogenic and antibiotic resistant strains with little representation of commensal strains and it is likely that not all isolates were tested for the aggregative phenotype. A larger, better defined, population of *E. coli* as a whole is needed to comprehensively define the distribution of EAEC in MLST complexes.

Although there are some MLST complexes/STs restricted to one country, these contain small numbers and all of the complexes with larger numbers of isolates are distributed throughout the phylogeny indicating a global distribution of the major clusters (Figure S2) most likely due to human travel. The independent appearance of the EAEC phenotype in discrete complexes across phylogeny (homoplasy), supports the observation of others [19] and suggests convergent evolution - the EAEC phenotype therefore confers a biological advantage in certain bacterial genetic backgrounds.

### Multiple genetic events have led to the independent evolution of EAEC

In order to understand the genetic events which led to the formation of different EAEC associated MLST complexes Clonal Frame analysis of the branching events for each node was carried out. Variation in the frequency of recombination or mutation which occurred in all of the seven loci at different time points was seen indicating multiple genetic events over time. The relative frequency of recombination as compared to mutation (ρ/θ) for the entire data set was 1.31 and is comparable to the rates proposed by Wirth et al [11] and Touchon et al [20] but higher than computed rates for the *E. coli* species via MLST including those that estimated recombination at approaching zero [21].

The parameters of rates and impact are based on the Markov model [22] which assumes that horizontal gene transfer events are equally probable between any pair of lineages, irrespective of phylogenetic and ecological proximity [23]. Our analysis clearly showed that this isn't the case and that (in this dataset) recombination rates vary within the EAEC pathotype between different lineages, the most ancestral being ST10 Cplx with the least impact of recombination in comparison to the other lineages (Table 5).

Multiple successful complexes (Figure 1) vary in mutation and recombination rate (Table 4) and are distributed throughout the *E. coli* population (Figure 2). These complexes have clearly evolved independently through multiple genetic events that have led to the phenotypic congruency of this pathotype. The selection of strains with a biological advantage has resulted in different, apparent, mutation/recombination rates suggests that certain bacterial backgrounds allow the advantage to be expressed - possibly influenced by the ability to retain the EAEC plasmid. Fast radiation of the complexes after population bottlenecks and frequent recombination seems a likely explanation for this pattern [11]. This may explain why the main gastrointestinal EAEC complexes were not found in the extra-intestinal *E. coli* phylogeny branch.

### Evolutionary events of EAEC

Although EAEC strains share the common phenotype of aggregative adherence, this and earlier research (Okeke et al 2010) demonstrates that the phenotype is convergent - has arisen in different lineages and been selected by survival in the human host. The selective advantage of aggregative adherence would allow *EAEC* strains to colonize the human gut during episodes of diarrhoea from other causes Lineages of EAEC found to be non-pathogenic are possibly strains that have developed exceptional colonization ability but not the ability to actually cause disease. Other lineages however, are associated with the ability to cause disease. Outbreak investigations and the strong association of some lineages with disease in this study point to multiple EAEC, but distinct, lineages that cause disease. Distinct sub-populations within a species may emerge because of differential local adaptation or genetic drift [14]. This concept may be applied to successful EAEC complexes which represent clusters of closely related genotypes and can be termed ecotypes [24] and will differ in their homologous recombination events because of adaptive evolution or environmental constraints [14]. This is supported by the variable recombination rate in different complexes which may have evolved from different environments. The variable recombination rate from each country will depend on the complexes found from the sample size tested. For EAEC isolates from UK residents the low impact of recombination may be because EAEC infection is related to travel and would therefore include EAEC found in multiple countries.

Virulent pathotypes have been shown to recombine more than non-pathogens pointing towards the theory that that virulence is the driving force for more frequent recombination [11]. This is shown with ST40 Cplx which is statistically associated with disease (p = 0.03) and had the highest impact of recombination on diversification. However ST10 Cplx, also statistically associated with disease (p = 0.01), had the highest rate of mutation among the complexes and the impact of recombination was almost equal to mutation (1∶1.2). This indicates that both types of genetic events are important in the evolution of pathogenic EAEC but that local variation occurs.

Our data analysis of the concatenated MLST sequences showed that the external to internal branch length ratio of the phylogeny was significantly higher than expected (Table 4). This means that the inferred genealogy is consistent with an expansion of the population size by acquisition of a fitness advantage early in the history of the sample [22]. For example, one suggestion is that the ancestral ST10 Cplx already had the background mutations to be able to acquire and retain the EAEC plasmid and so the external to internal branch length ratio is as expected. This fits in with previous studies where a specific genetic background is required to acquire and express virulence factors in *E. coli* [25]. Other complexes with unexpected external to internal branch length ratio, such as ST40 Cplx, needed recombination and/or mutation events to allow the stable retention of the advantageous EAEC plasmid. A recently reported example of how acquisition of this EAEC plasmid can increase fitness is the ST678 (O104) VTEC German outbreak [26]. This is a VTEC strain that didn't have the characteristic *eae* gene (attachment and effacement loci for intimate adherence) but did have the plasmid encoded *aat* gene cluster associated with adherence. This strain was particularly virulent, with high HUS rates, but had the same toxin type as many other VTEC strains, the difference, presumably, being its strong ability to adhere and hence introduce more toxin. This basic mechanism of attachment could be the fitness advantage that this relatively new pathotype, EAEC, has harboured and then successfully expanded.

## Conclusions

This study has clearly shown the complexity of the evolution of EAEC, while it is evident that the same lineages prevail in multiple global locations, indicative of clonal expansion, whilst other lineages are ecologically adapting through a process of convergent evolution. This would account for the inconsistent impact rates of recombination between different geographical locations and different complexes. The collection of organisms given the “pathotype” EAEC has evolved as multiple independent lineages with some complexes associated with disease, but not all. This is important as a non-disease causing EAEC still has the ability to acquire other virulence factors and the combination of aggregative adherence and virulence can cause severe outbreaks. The presence of the *aggR* genes as an indicator of aggregative adherence ability should therefore be considered when diagnosing gastrointestinal disease.

## Supporting Information

Figure S1 **Minimal Spanning Tree of 443 enteroaggregative** ***E. coli*** **serotyped.** Minimum Spanning Tree of 443 EAEC serotyped in this study. Tree is colour coded by serotypes containing 3 or more isolates. Serotypes shown in one or two strains were coloured white. Complexes shaded in grey consist of single locus variants (SLV). Sequence types are labelled as numbers.
(TIF)

Figure S2 **MSTree Geographical location.** Minimal spanning tree of the 564 EAEC used in this study colour coded by isolates from Bangladesh (red), Nigeria (purple) and UK (green) and the prototypical O42 strain from Peru (yellow). Complexes shaded in grey consist of single locus variants (SLV). Trees shows that complexes are mainly distrusted in at least two countries with only a few small complexes and singletons geographically specific. Sequence types and complex (Cplx) are labelled as numbers.
(TIF)

Table S1 **Strain list used in this study.** Table of strains used in this study listing the year the strain was isolated, the Country the strain was isolated from, somatic and flagella typing results (serotyping), sequence type and complex the strain belongs to. NT: Not tested, Novel sequence types consisted of either single locus variants (SLV), double locus variants (DLV) or triple locus variants (TLV) of known sequence types.
(PDF)
