# Supplementary figures and images for: Enteroaggregative Escherichia coli Have Evolved Independently as Distinct Complexes within the E. coli Population with Varying Ability to Cause Disease
Source: PLoS One. 2014 Nov 21;9(11):e112967. doi: 10.1371/journal.pone.0112967 (PMC4240581; doi:10.1371/journal.pone.0112967)

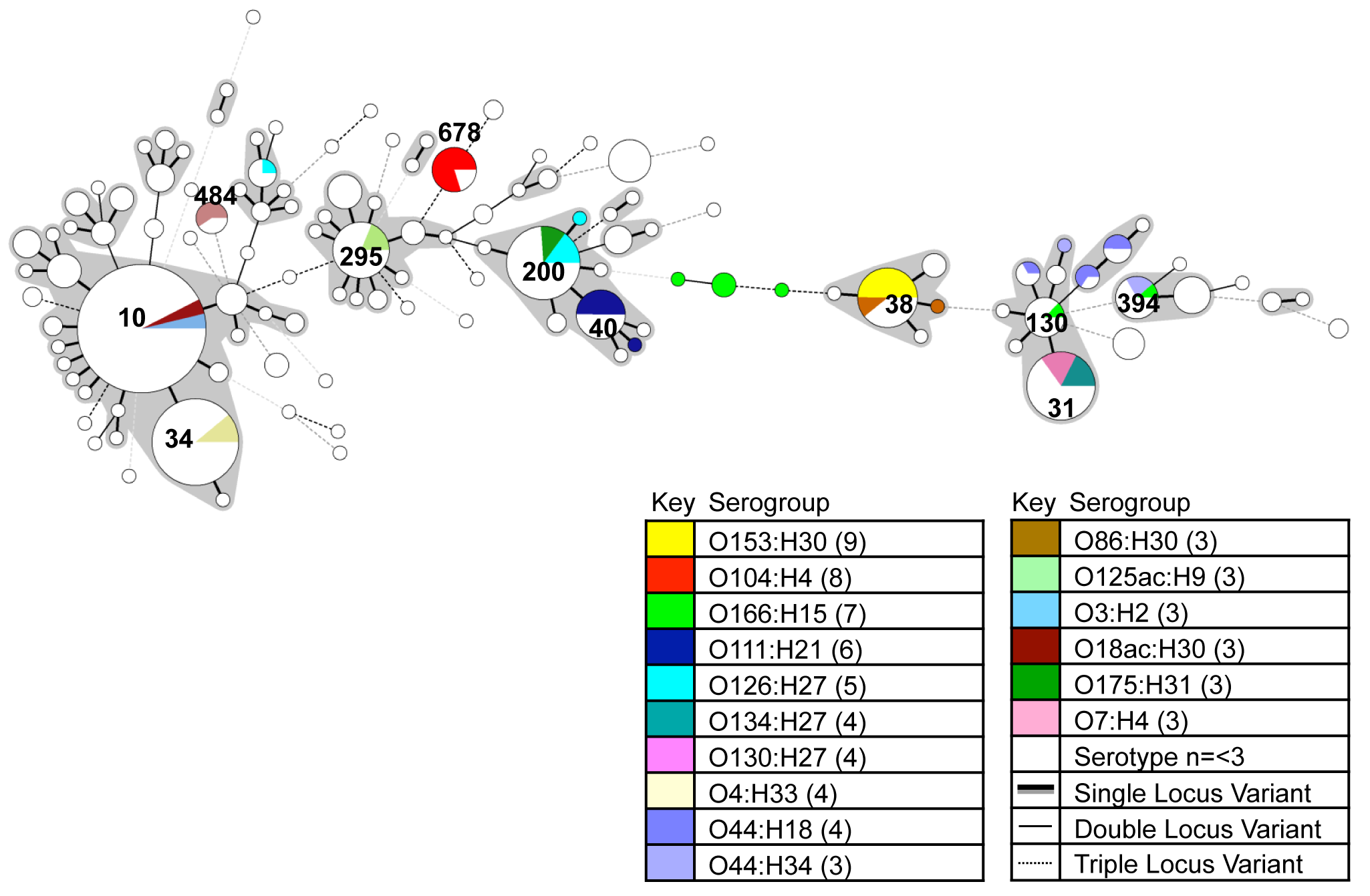

Supplement: Figure S1 — Minimal Spanning Tree of 443 enteroaggregative E. coli serotyped. Minimum Spanning Tree of 443 EAEC serotyped in this study. Tree is colour coded by serotypes containing 3 or more isolates. Serotypes shown in one or two strains were coloured white. Complexes shaded in grey consist of single locus variants (SLV). Sequence types are labelled as numbers. (TIF) [file pone.0112967.s001.tif]

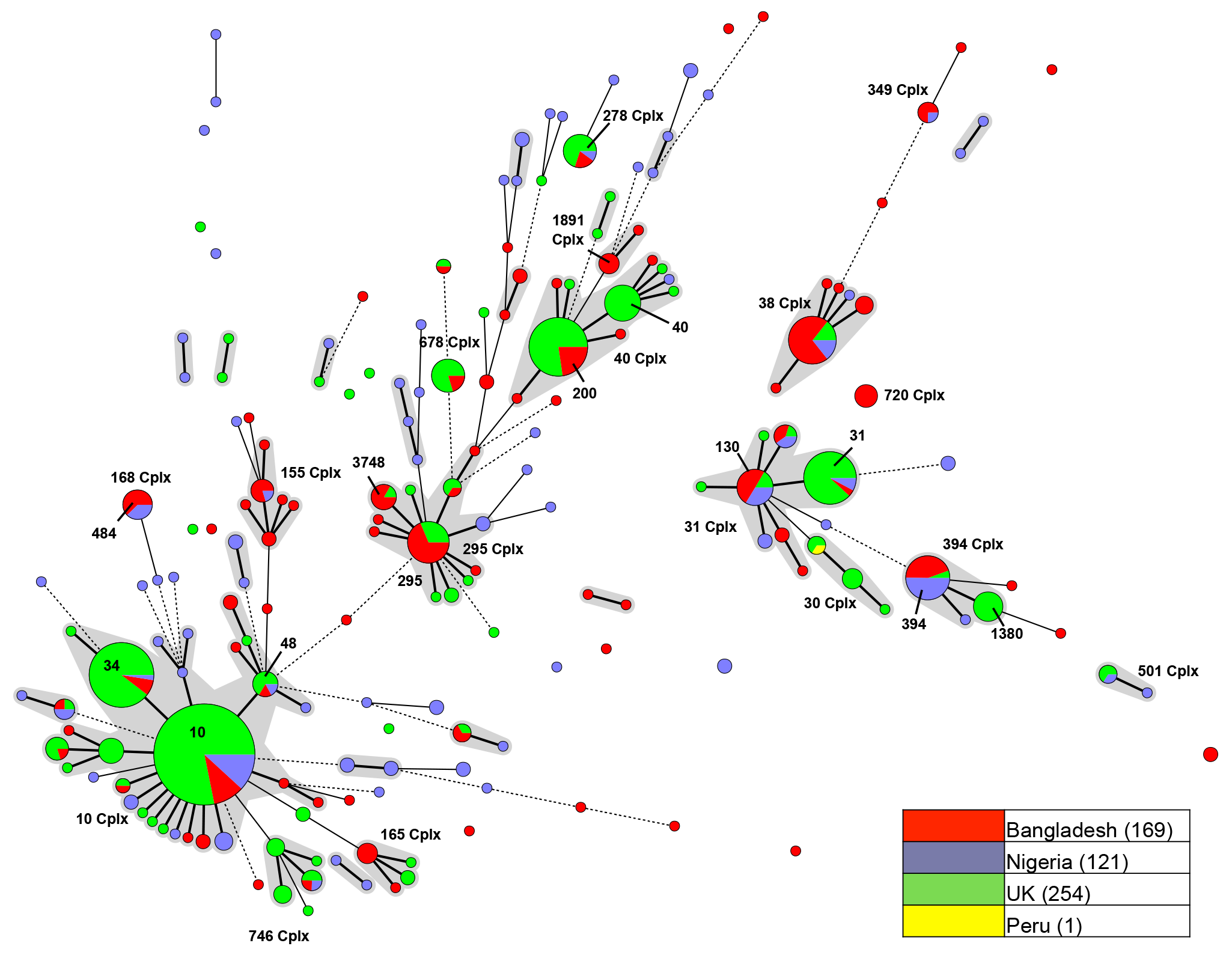

Supplement: Figure S2 — MSTree Geographical location. Minimal spanning tree of the 564 EAEC used in this study colour coded by isolates from Bangladesh (red), Nigeria (purple) and UK (green) and the prototypical O42 strain from Peru (yellow). Complexes shaded in grey consist of single locus variants (SLV). Trees shows that complexes are mainly distrusted in at least two countries with only a few small complexes and singletons geographically specific. Sequence types and complex (Cplx) are labelled as numbers. (TIF) [file pone.0112967.s002.tif]
