# Supplementary material for: Enteroaggregative Escherichia coli Have Evolved Independently as Distinct Complexes within the E. coli Population with Varying Ability to Cause Disease
Source: PLoS One. 2014 Nov 21;9(11):e112967. doi: 10.1371/journal.pone.0112967 (PMC4240581; doi:10.1371/journal.pone.0112967)
Supplement: Table S1 — Strain list used in this study. Table of strains used in this study listing the year the strain was isolated, the Country the strain was isolated from, somatic and flagella typing results (serotyping), sequence type and complex the strain belongs to. NT: Not tested, Novel sequence types consisted of either single locus variants (SLV), double locus variants (DLV) or triple locus variants (TLV) of known sequence types. (PDF) [file pone.0112967.s003.pdf]

Isolate	Year	Country	Pathogen	Somatic	Flagella	ST	ST Complex
1024	2007-2011	Bangladesh	Case	130	27	10	ST10 Cplx
1116	2007-2011	Bangladesh	Case	O?	33	10	ST10 Cplx
3042	2007-2011	Bangladesh	Case	3	2	10	ST10 Cplx
601051	2007-2011	Bangladesh	Control	154	19	10	ST10 Cplx
601090	2007-2011	Bangladesh	Case	O?	33	10	ST10 Cplx
601091	2007-2011	Bangladesh	Case	O?	H-	10	ST10 Cplx
601096	2007-2011	Bangladesh	Case	O?	33	10	ST10 Cplx
601197	2007-2011	Bangladesh	Control	3	41	10	ST10 Cplx
E89111	1993	Bangladesh	Case	89	18	10	ST10 Cplx
17-2/ E92356	1994	Chilie	Case	3	2	10	ST10 Cplx
G49	1995	Nigeria	Case	NT	NT	10	ST10 Cplx
C10	1995	Nigeria	Case	NT	NT	10	ST10 Cplx
G04a	1995	Nigeria	Case	NT	NT	10	ST10 Cplx
D25a	1995	Nigeria	Case	NT	NT	10	ST10 Cplx
C14a	1995	Nigeria	Case	NT	NT	10	ST10 Cplx
C17	1995	Nigeria	Case	NT	NT	10	ST10 Cplx
G63	1995	Nigeria	Case	NT	NT	10	ST10 Cplx
C55	1995	Nigeria	Control	NT	NT	10	ST10 Cplx
G133	1995	Nigeria	Control	NT	NT	10	ST10 Cplx
G123	1995	Nigeria	Control	NT	NT	10	ST10 Cplx
G129	1995	Nigeria	Control	NT	NT	10	ST10 Cplx
2278	2008	UK	Case	3	2	10	ST10 Cplx
E099963	1994	UK	Case	18ac	30	10	ST10 Cplx
E099964	1994	UK	Case	18ac	H-	10	ST10 Cplx
E099969	1994	UK	Control	134	H-	10	ST10 Cplx
E099970	1994	UK	Case	53	H-	10	ST10 Cplx
E099971	1994	UK	Control	53	2	10	ST10 Cplx
E099974	1994	UK	Case	O?	H?	10	ST10 Cplx
E099977	1994	UK	Case	86	H-	10	ST10 Cplx
E100866	1994	UK	Case	7	2	10	ST10 Cplx
E100875	1994	UK	Case	7	2	10	ST10 Cplx
E101092	1994	UK	Case	O?	H?	10	ST10 Cplx
E101097	1994	UK	Case	O?	H?	10	ST10 Cplx
E103599	1994	UK	Case	O?	H?	10	ST10 Cplx
E103619	1994	UK	Case	O?	H?	10	ST10 Cplx
E104927	1994	UK	Control	O?	H?	10	ST10 Cplx
E104965	1994	UK	Case	O?	H?	10	ST10 Cplx
E104966	1994	UK	Case	O?	H?	10	ST10 Cplx
E104968	1994	UK	Case	131	H?	10	ST10 Cplx
E106505	1994	UK	Case	O?	H?	10	ST10 Cplx
E106776	1994	UK	Control	O?	H?	10	ST10 Cplx
E107246	1994	UK	Control	O?	H?	10	ST10 Cplx
E107248	1994	UK	Case	O?	H?	10	ST10 Cplx
E107249	1994	UK	Case	21	4	10	ST10 Cplx
E107525	1994	UK	Case	O?	H?	10	ST10 Cplx

E107536	1994	UK	Case	O?	H?	10	ST10 Cplx
E107537	1994	UK	Case	O?	H?	10	ST10 Cplx
E107541	1994	UK	Case	O?	H?	10	ST10 Cplx
E107743	1994	UK	Case	O?	H?	10	ST10 Cplx
E107755	1995	UK	Control	21	H-	10	ST10 Cplx
E094712	1994	UK	Case	3	H-	10	ST10 Cplx
E096616	1994	UK	Control	5	4	10	ST10 Cplx
E097477	1994	UK	Case	81	H-	10	ST10 Cplx
E097494	1994	UK	Case	81	H-	10	ST10 Cplx
E097496	1994	UK	Case	86	2	10	ST10 Cplx
E097499	1994	UK	Case	6	10	10	ST10 Cplx
E099535	1994	UK	Case	R	27	10	ST10 Cplx
E099959	1994	UK	Case	66	25	10	ST10 Cplx
E099960	1994	UK	Case	18ac	30	10	ST10 Cplx
E099961	1994	UK	Control	18ac	30	10	ST10 Cplx
E108398	1994	UK	Case	O?	H?	10	ST10 Cplx
E108692	1995	UK	Control	O?	H?	10	ST10 Cplx
E108838	1995	UK	Case	O?	H?	10	ST10 Cplx
E109634	1995	UK	Control	O?	H?	10	ST10 Cplx
E109903	1995	UK	Case	21	H-	10	ST10 Cplx
E109906	1995	UK	Case	O?	H?	10	ST10 Cplx
E110899	1995	UK	Case	O?	H?	10	ST10 Cplx
E111139	1995	UK	Case	4	2	10	ST10 Cplx
E111260	1995	UK	Control	4	2	10	ST10 Cplx
E111479	1995	UK	Control	11	H-	10	ST10 Cplx
E59905	1989	UK	Case	21	2	10	ST10 Cplx
E67643	1990	UK	Case	78	10	10	ST10 Cplx
E97470	1994	UK	Case	86	34	10	ST10 Cplx
13540517	2011	UK	Case	O?	H-	10	ST10 Cplx
23600287	2012	UK	Case	168	4	10	ST10 Cplx
23600288	2012	UK	Case	168	4	10	ST10 Cplx
23620421	2012	UK	Case	99	52	10	ST10 Cplx
31440284	2013	UK	Case	O?	H?	10	ST10 Cplx
31720375	2013	UK	Case	99	H?	10	ST10 Cplx
31720376	2013	UK	Case	100	19	10	ST10 Cplx
31720388	2013	UK	Case	101	5	10	ST10 Cplx
32360076	2013	UK	Case	175	H?	10	ST10 Cplx
31920219	2013	UK	Case	131	27	10	ST10 Cplx
31920220	2013	UK	Case	131	27	10	ST10 Cplx
31941061	2013	UK	Case	55	19	10	ST10 Cplx
31941071	2013	UK	Case	131	27	10	ST10 Cplx
31941072	2013	UK	Case	131	27	10	ST10 Cplx
31941073	2013	UK	Case	131	27	10	ST10 Cplx
31941074	2013	UK	Case	131	27	10	ST10 Cplx
2336	2008	UK	Case	114	10	10	ST10 Cplx
2633	2008	UK	Case	O?	1	10	ST10 Cplx

2745	2008	UK	Case	114	10	10	ST10 Cplx
G26a	1995	Nigeria	Case	NT	NT	23	ST23 Cplx
E100877	1994	UK	Case	O?	H?	30	ST30 Cplx
E103267	1994	UK	Case	O?	H?	30	ST30 Cplx
E44939	1987	UK	Case	44	18	30	ST30 Cplx
E45730	1987	UK	Case	44	18	30	ST30 Cplx
601120	2007-2011	Bangladesh	Control	130	27	31	ST31 Cplx
E54	1995	Nigeria	Control	NT	NT	31	ST31 Cplx
E56	1994	Nigeria	Control	NT	NT	31	ST31 Cplx
E099975	1994	UK	Control	15	18	31	ST31 Cplx
E101091	1994	UK	Case	134	27	31	ST31 Cplx
E101095	1994	UK	Case	134	2	31	ST31 Cplx
E101096	1994	UK	Case	134	27	31	ST31 Cplx
E103594	1994	UK	Case	11	27	31	ST31 Cplx
E103617	1994	UK	Case	130	27	31	ST31 Cplx
E104931	1994	UK	Control	O?	H?	31	ST31 Cplx
E104940	1994	UK	Control	130	27	31	ST31 Cplx
E104946	1994	UK	Case	O?	H?	31	ST31 Cplx
E104967	1994	UK	Case	O?	H?	31	ST31 Cplx
E104969	1994	UK	Control	130	25	31	ST31 Cplx
E107526	1994	UK	Case	O?	H?	31	ST31 Cplx
E107754	1995	UK	Control	134	25	31	ST31 Cplx
E097478	1994	UK	Control	130	27	31	ST31 Cplx
E097502	1994	UK	Case	134	27	31	ST31 Cplx
E108837	1995	UK	Control	130	H?	31	ST31 Cplx
E108839	1995	UK	Case	130	27	31	ST31 Cplx
E110852	1995	UK	Case	O?	H?	31	ST31 Cplx
E111136	1995	UK	Control	1	H-	31	ST31 Cplx
E71341	1991	UK	Case	102	27	31	ST31 Cplx
E96485	1994	UK	Case	134	27	31	ST31 Cplx
E98529	1994	UK	Case	O?	18	31	ST31 Cplx
3074	2007-2011	Bangladesh	Control	56	48	34	ST10 Cplx
900008	2007-2011	Bangladesh	Case	O?	10	34	ST10 Cplx
601134	2007-2011	Bangladesh	Control	O?	10	34	ST10 Cplx
G170	1995	Nigeria	Control	NT	NT	34	ST10 Cplx
E100868	1994	UK	Case	4	33	34	ST10 Cplx
E100870	1994	UK	Case	R	H?	34	ST10 Cplx
E100872	1994	UK	Case	62	30	34	ST10 Cplx
E100876	1994	UK	Case	O?	H?	34	ST10 Cplx
E101093	1994	UK	Case	O?	H?	34	ST10 Cplx
E101094	1994	UK	Case	4	33	34	ST10 Cplx
E104942	1994	UK	Case	162	10	34	ST10 Cplx
E104972	1994	UK	Case	62	30	34	ST10 Cplx
E104973	1994	UK	Case	O?	H?	34	ST10 Cplx
E105355	1994	UK	Case	O?	H?	34	ST10 Cplx
E105389	1994	UK	Case	O?	H?	34	ST10 Cplx

E107241	1994	UK	Case	O?	H?	34	ST10 Cplx
E107528	1994	UK	Case	O?	H?	34	ST10 Cplx
E107529	1994	UK	Case	O?	H?	34	ST10 Cplx
E092843	1993	UK	Case	O?	H?	34	ST10 Cplx
E096612	1994	UK	Case	R	H?	34	ST10 Cplx
E099536	1994	UK	Case	4	33	34	ST10 Cplx
E108400	1994	UK	Case	O?	H?	34	ST10 Cplx
E108689	1995	UK	Control	O?	H?	34	ST10 Cplx
E108690	1995	UK	Case	O?	H?	34	ST10 Cplx
E109902	1995	UK	Control	4	33	34	ST10 Cplx
E111262	1995	UK	Case	O?	H?	34	ST10 Cplx
E111294	1995	UK	Control	O?	H?	34	ST10 Cplx
E112215	1995	UK	Case	O?	H?	34	ST10 Cplx
E60725	1990	UK	Case	92	33	34	ST10 Cplx
E73339	1991	UK	Case	15	H-	34	ST10 Cplx
E96483	1994	UK	Case	O?	33	34	ST10 Cplx
E96487	1994	UK	Case	O?	H-	34	ST10 Cplx
E97820	1994	UK	Case	62	30	34	ST10 Cplx
E97900	1994	UK	Case	O?	H-	34	ST10 Cplx
1360104	2010	UK	Case	O?	H?	34	ST10 Cplx
32320086	2013	UK	Case	92	H?	34	ST10 Cplx
2451	2008	UK	Case	O?	33	34	ST10 Cplx
2466	2008	UK	Case	92	33	34	ST10 Cplx
7016	2007-2011	Bangladesh	Control	153	30	38	ST38 Cplx
900033	2007-2011	Bangladesh	Case	153	30	38	ST38 Cplx
900516	2007-2011	Bangladesh	Case	86	30	38	ST38 Cplx
900252	2007-2011	Bangladesh	Case	153	30	38	ST38 Cplx
900002	2007-2011	Bangladesh	Case	153	30	38	ST38 Cplx
900745	2007-2011	Bangladesh	Case	153	30	38	ST38 Cplx
601000	2007-2011	Bangladesh	Control	O?	30	38	ST38 Cplx
601029	2007-2011	Bangladesh	Case	O?	32	38	ST38 Cplx
601070	2007-2011	Bangladesh	Case	O?	30	38	ST38 Cplx
601108	2007-2011	Bangladesh	Control	153	30	38	ST38 Cplx
601182	2007-2011	Bangladesh	Control	O?	30	38	ST38 Cplx
601225	2007-2011	Bangladesh	Control	86	30	38	ST38 Cplx
601264	2007-2011	Bangladesh	Case	153	30	38	ST38 Cplx
7060	2007-2011	Bangladesh	Case	O?	27	38	ST38 Cplx
7002	2007-2011	Bangladesh	Control	181	36	38	ST38 Cplx
G75a	1995	Nigeria	Case	NT	NT	38	ST38 Cplx
G23	1995	Nigeria	Case	NT	NT	38	ST38 Cplx
G29	1995	Nigeria	Case	NT	NT	38	ST38 Cplx
32760800	2013	UK	Case	O?	H?	38	ST38 Cplx
669	2008	UK	Case	153	30	38	ST38 Cplx
1975	2008	UK	Case	153	30	38	ST38 Cplx
1150	2008	UK	Case	111ac	21	40	ST40 Cplx
E107759	1995	UK	Control	O?	H?	40	ST40 Cplx

E109907	1995	UK	Control	111ab	H-	40	ST40 Cplx
E36182	1984	UK	Case	111	21	40	ST40 Cplx
4680397	2010	UK	Case	111ab	21	40	ST40 Cplx
20820356	2012	UK	Case	111ab	21	40	ST40 Cplx
22840058	2012	UK	Case	111ab	11	40	ST40 Cplx
20680226	2012	UK	Case	111ab	21	40	ST40 Cplx
657	2008	UK	Case	111ac	11	40	ST40 Cplx
1091	2008	UK	Case	111ac	11	40	ST40 Cplx
1244	2008	UK	Case	111ac	21	40	ST40 Cplx
1337	2008	UK	Case	111ac	11	40	ST40 Cplx
E103597	1994	UK	Case	R	H?	43	ST10 Cplx
E104943	1994	UK	Control	O?	H?	43	ST10 Cplx
E105837	1994	UK	Control	O?	H?	43	ST10 Cplx
E107533	1994	UK	Case	O?	H?	43	ST10 Cplx
E109635	1995	UK	Case	O?	H?	43	ST10 Cplx
E110715	1995	UK	Case	6	10	43	ST10 Cplx
G57	1995	Nigeria	Case	NT	NT	46	ST46 Cplx
600978	2007-2011	Bangladesh	Case	O?	11	48	ST10 Cplx
G41	1995	Nigeria	Case	NT	NT	48	ST10 Cplx
E099966	1994	UK	Case	151	11	48	ST10 Cplx
E110910	1995	UK	Case	118	11	48	ST10 Cplx
E97590	1994	UK	Case	73	13	48	ST10 Cplx
651	2008	UK	Case	151	21	48	ST10 Cplx
G37a	1995	Nigeria	Case	NT	NT	52	None
E89105	1993	Bangladesh	Case	80	27	58	ST155 Cplx
8192	2007-2011	Bangladesh	Case	O?	19	58	ST155 Cplx
E107527	1994	UK	Control	6	1	73	ST73 Cplx
7040	2007-2011	Bangladesh	Case	60	22	101	ST101 Cplx
E89104	1993	Bangladesh	Case	141	49	111	None
E099534	1994	UK	Case	86	34	117	None
2506	2008	UK	Case	153	2	120	None
900422	2007-2011	Bangladesh	Case	176	34	130	ST31 Cplx
900268	2007-2011	Bangladesh	Case	44	34	130	ST31 Cplx
900985	2007-2011	Bangladesh	Case	O?	23	130	ST31 Cplx
600988	2007-2011	Bangladesh	Case	166	16	130	ST31 Cplx
601048	2007-2011	Bangladesh	Control	O?	23	130	ST31 Cplx
901006	2007-2011	Bangladesh	Case	44	34	130	ST31 Cplx
C20	1995	Nigeria	Case	NT	NT	130	ST31 Cplx
G22b	1995	Nigeria	Case	NT	NT	130	ST31 Cplx
C60	1995	Nigeria	Control	NT	NT	130	ST31 Cplx
G116	1995	Nigeria	Control	NT	NT	130	ST31 Cplx
1383	2008	UK	Case	44	12	130	ST31 Cplx
E105352	1994	UK	Control	O?	H?	130	ST31 Cplx
D31b	1995	Nigeria	Case	NT	NT	144	None
22780069	2012	UK	Case	O?	H?	152	ST152 Cplx
900098	2007-2011	Bangladesh	Case	126	27	155	ST155 Cplx

601191	2007-2011	Bangladesh	Case	9	21	155	ST155 Cplx
601307	2007-2011	Bangladesh	Case	9	21	155	ST155 Cplx
8225	2007-2011	Bangladesh	Control	15	34	155	ST155 Cplx
G48	1995	Nigeria	Case	NT	NT	155	ST155 Cplx
7064	2007-2011	Bangladesh	Control	153	36	157	None
900512	2007-2011	Bangladesh	Case	O?	23	157	None
E89096	1993	Bangladesh	Case	113	H-	159	ST746 Cplx
C27	1995	Nigeria	Case	NT	NT	159	ST746 Cplx
E107758	1995	UK	Case	O?	H?	159	ST746 Cplx
32100889	2013	UK	Case	181	H?	159	ST746 Cplx
900547	2007-2011	Bangladesh	Case	O?	19	165	ST165 Cplx
900603	2007-2011	Bangladesh	Case	O?	19	165	ST165 Cplx
900820	2007-2011	Bangladesh	Case	O?	19	165	ST165 Cplx
7081	2007-2011	Bangladesh	Control	O?	19	165	ST165 Cplx
G36	1995	Nigeria	Case	NT	NT	167	ST10 Cplx
G85	1995	Nigeria	Case	NT	NT	167	ST10 Cplx
C53	1995	Nigeria	Control	NT	NT	167	ST10 Cplx
900657	2007-2011	Bangladesh	Case	175	28	200	ST40 Cplx
900618	2007-2011	Bangladesh	Case	175	7	200	ST40 Cplx
601033	2007-2011	Bangladesh	Control	175	31	200	ST40 Cplx
601192	2007-2011	Bangladesh	Case	175	1	200	ST40 Cplx
601193	2007-2011	Bangladesh	Control	175	31	200	ST40 Cplx
900987	2007-2011	Bangladesh	Case	175	31	200	ST40 Cplx
900998	2007-2011	Bangladesh	Case	175	1	200	ST40 Cplx
E40104	1985	UK	Case	126	H?	200	ST40 Cplx
1171	2008	UK	Case	126	27	200	ST40 Cplx
E099967	1994	UK	Case	R	H?	200	ST40 Cplx
E099972	1994	UK	Case	O?	H?	200	ST40 Cplx
E099979	1994	UK	Case	75	27	200	ST40 Cplx
E100856	1994	UK	Case	126	27	200	ST40 Cplx
E101089	1994	UK	Case	126	2	200	ST40 Cplx
E106506	1994	UK	Case	O?	H?	200	ST40 Cplx
E107100	1994	UK	Case	119	27	200	ST40 Cplx
E107531	1994	UK	Case	R	H?	200	ST40 Cplx
E107542	1994	UK	Case	R	H?	200	ST40 Cplx
E097298	1994	UK	Case	R	27	200	ST40 Cplx
E097501	1994	UK	Case	O?	H?	200	ST40 Cplx
E099520	1994	UK	Case	8	7	200	ST40 Cplx
E110717	1995	UK	Case	O?	H?	200	ST40 Cplx
E111140	1995	UK	Case	O?	H?	200	ST40 Cplx
E43923	1987	UK	Case	126	27	200	ST40 Cplx
E55060	1988	UK	Case	126	27	200	ST40 Cplx
23160613	2012	UK	Case	175	28	200	ST40 Cplx
23520278	2012	UK	Case	175	28	200	ST40 Cplx
23520279	2012	UK	Case	175	28	200	ST40 Cplx
24020363	2012	UK	Case	175	31	200	ST40 Cplx

32360372	2013	UK	Case	126	27	200	ST40 Cplx
2266	2008	UK	Case	175	27	200	ST40 Cplx
600983	2007-2011	Bangladesh	Control	60	H?	206	ST206 Cplx
8098	2007-2011	Bangladesh	Control	11	16	206	ST206 Cplx
4480153	2010	UK	Case	O?	H?	206	ST206 Cplx
C29	1995	Nigeria	Case	NT	NT	218	ST10 Cplx
G50	1995	Nigeria	Case	NT	NT	218	ST10 Cplx
900694	2007-2011	Bangladesh	Case	O?	24	219	None
900157	2007-2011	Bangladesh	Case	34	11	223	ST155 Cplx
7207	2007-2011	Bangladesh	Case	91	9	226	ST226 Cplx
G35	1995	Nigeria	Case	NT	NT	226	ST226 Cplx
E53	1995	Nigeria	Control	NT	NT	226	ST226 Cplx
31941060	2013	UK	Case	111ac	4	226	ST226 Cplx
31941064	2013	UK	Case	O?	21	227	ST10 Cplx
E89107	1993	Bangladesh	Case	O?	27	278	ST278 Cplx
E89115	1993	Bangladesh	Case	162	H-	278	ST278 Cplx
D29	1995	Nigeria	Case	NT	NT	278	ST278 Cplx
E104970	1994	UK	Case	33	H-	278	ST278 Cplx
E101396	1995	UK	Case	98	H-	278	ST278 Cplx
E101406	1995	UK	Case	98	H-	278	ST278 Cplx
E101621	1994	UK	Case	98	H-	278	ST278 Cplx
E111613	1995	UK	Control	165	4	278	ST278 Cplx
31920215	2013	UK	Case	20	H?	278	ST278 Cplx
31920221	2013	UK	Case	20	19	278	ST278 Cplx
8080	2007-2011	Bangladesh	Case	125ac	9	295	ST295 Cplx
900286	2007-2011	Bangladesh	Case	O?	27	295	ST295 Cplx
601083	2007-2011	Bangladesh	Control	O?	28	295	ST295 Cplx
601144	2007-2011	Bangladesh	Control	125ac	9	295	ST295 Cplx
601188	2007-2011	Bangladesh	Control	181	3	295	ST295 Cplx
601251	2007-2011	Bangladesh	Case	O?	29	295	ST295 Cplx
7058	2007-2011	Bangladesh	Control	O?	16	295	ST295 Cplx
7078	2007-2011	Bangladesh	Control	O?	27	295	ST295 Cplx
7096	2007-2011	Bangladesh	Control	O?	12	295	ST295 Cplx
8002	2007-2011	Bangladesh	Control	9a	10	295	ST295 Cplx
8129	2007-2011	Bangladesh	Control	O?	29	295	ST295 Cplx
E104974	1994	UK	Case	O?	H?	295	ST295 Cplx
E092836	1993	UK	Case	O?	H?	295	ST295 Cplx
E58596	1989	UK	Case	51	11	295	ST295 Cplx
31920217	2013	UK	Case	33	16	295	ST295 Cplx
1778	2008	UK	Case	125ac	9	295	ST295 Cplx
900978	2007-2011	Bangladesh	Case	O?	30	315	ST38 Cplx
900912	2007-2011	Bangladesh	Case	O?	30	315	ST38 Cplx
7123	2007-2011	Bangladesh	Control	21	10	315	ST38 Cplx
23630425	2012	UK	Case	136	54	329	None
3017	2007-2011	Bangladesh	Control	166	15	349	ST349 Cplx
600950	2007-2011	Bangladesh	Control	166	15	349	ST349 Cplx

600990	2007-2011	Bangladesh	Case	166	15	349	ST349 Cplx
E60	1995	Nigeria	Control	NT	NT	349	ST349 Cplx
E54a	1995	Nigeria	Control	NT	NT	362	None
E72	1995	Nigeria	Control	NT	NT	362	None
900673	2007-2011	Bangladesh	Case	O?	18	394	ST394 Cplx
900088	2007-2011	Bangladesh	Case	44	40	394	ST394 Cplx
900416	2007-2011	Bangladesh	Case	O?	18	394	ST394 Cplx
600970	2007-2011	Bangladesh	Case	O?	18	394	ST394 Cplx
601002	2007-2011	Bangladesh	Control	O?	41	394	ST394 Cplx
601009	2007-2011	Bangladesh	Case	166	15	394	ST394 Cplx
601230	2007-2011	Bangladesh	Control	44	18	394	ST394 Cplx
E89102	1993	Bangladesh	Case	44	18	394	ST394 Cplx
G59	1995	Nigeria	Case	NT	NT	394	ST394 Cplx
G10	1995	Nigeria	Case	NT	NT	394	ST394 Cplx
C14	1995	Nigeria	Case	NT	NT	394	ST394 Cplx
G17a	1995	Nigeria	Case	NT	NT	394	ST394 Cplx
C08	1995	Nigeria	Case	NT	NT	394	ST394 Cplx
E30	1995	Nigeria	Case	NT	NT	394	ST394 Cplx
G67b	1995	Nigeria	Case	NT	NT	394	ST394 Cplx
G108	1995	Nigeria	Control	NT	NT	394	ST394 Cplx
E64	1995	Nigeria	Control	NT	NT	394	ST394 Cplx
E106507	1994	UK	Case	O?	H?	394	ST394 Cplx
JPN10	1985	Peru	Case	44	18	414	ST30 Cplx
E110716	1995	UK	Case	106	18	414	ST30 Cplx
E43509	1987	UK	Case	44	18	414	ST30 Cplx
G52	1995	Nigeria	Case	NT	NT	422	None
G55	1995	NIgeria	Case	NT	NT	423	ST23 Cplx
G74	1995	Nigeria	Case	NT	NT	424	None
G04	1995	Nigeria	Case	NT	NT	425	None
G28	1995	Nigeria	Case	NT	NT	426	ST38 Cplx
G02	1995	Nigeria	Case	NT	NT	433	ST295 Cplx
G131	1995	Nigeria	Control	NT	NT	433	ST295 Cplx
E16	1995	Nigeria	Case	NT	NT	434	None
G01	1995	Nigeria	Case	NT	NT	435	None
E10	1995	Nigeria	Case	NT	NT	435	None
C22	1995	Nigeria	Case	NT	NT	436	ST10 Cplx
C05a	1995	Nigeria	Case	NT	NT	437	None
G30a	1995	Nigeria	Case	NT	NT	438	None
C61	1995	Nigeria	Control	NT	NT	444	ST446 Cplx
900644	2007-2011	Bangladesh	Case	O?	10	448	ST448 Cplx
601063	2007-2011	Bangladesh	Control	15	18	449	ST31 Cplx
E89114	1993	Bangladesh	Case	44	18	449	ST31 Cplx
E68	1995	Nigeria	Control	NT	NT	449	ST31 Cplx
G103	1995	Nigeria	Control	NT	NT	449	ST31 Cplx
E108693	1995	UK	Control	O?	H?	449	ST31 Cplx
G106	1995	Nigeria	Control	NT	NT	450	None

E25	1995	Nigeria	Case	NT	NT	453	ST86 Cplx
G16	1995	Nigeria	Case	NT	NT	454	None
G05b	1995	Nigeria	Case	NT	NT	455	None
C66	1995	Nigeria	Control	NT	NT	455	None
E59	1995	Nigeria	Control	NT	NT	456	None
G107	1995	Nigeria	Control	NT	NT	456	None
C54a	1995	Nigeria	Control	NT	NT	459	None
C54b	1995	Nigeria	Control	NT	NT	459	None
D09	1995	Nigeria	Case	NT	NT	460	ST168 Cplx
G137	1995	Nigeria	Control	NT	NT	461	ST23 Cplx
D08	1995	Nigeria	Case	NT	NT	464	None
C68	1995	Nigeria	Control	NT	NT	466	ST23 Cplx
C70	1995	Nigeria	Control	NT	NT	467	ST467 Cplx
G143	1995	Nigeria	Control	NT	NT	467	ST467 Cplx
C78a	1995	Nigeria	Control	NT	NT	468	None
C77	1995	Nigeria	Control	NT	NT	469	ST469 Cplx
E66	1995	Nigeria	Control	NT	NT	470	None
E62	1995	Nigeria	Control	NT	NT	471	ST394 Cplx
E63	1995	Nigeria	Control	NT	NT	473	ST40 Cplx
G149	1994	Nigeria	Control	NT	NT	474	ST31 Cplx
G121a	1995	Nigeria	Control	NT	NT	474	ST31 Cplx
G126	1995	Nigeria	Control	NT	NT	475	ST10 Cplx
E74	1995	Nigeria	Control	NT	NT	476	ST12 Cplx
G159	1995	Nigeria	Control	NT	NT	477	ST226 Cplx
G12a	1995	Nigeria	Case	NT	NT	478	ST206 Cplx
D34	1995	Nigeria	Case	NT	NT	480	ST467 Cplx
D32	1995	Nigeria	Control	NT	NT	480	ST467 Cplx
D34a	1995	Nigeria	Case	NT	NT	481	None
D33b	1995	Nigeria	Case	NT	NT	481	None
G146	1995	Nigeria	Control	NT	NT	483	None
3029	2007-2011	Bangladesh	Control	12	4	484	ST168 Cplx
900696	2007-2011	Bangladesh	Case	7	4	484	ST168 Cplx
601235	2007-2011	Bangladesh	Case	7	4	484	ST168 Cplx
8089	2007-2011	Bangladesh	Control	7	4	484	ST168 Cplx
7172	2007-2011	Bangladesh	Control	12	4	484	ST168 Cplx
E33	1995	Nigeria	Case	NT	NT	484	ST168 Cplx
G30	1995	Nigeria	Case	NT	NT	484	ST168 Cplx
G110	1995	Nigeria	Control	NT	NT	484	ST168 Cplx
E07	1995	Nigeria	Case	NT	NT	485	None
C28	1995	Nigeria	Case	NT	NT	486	None
E12	1995	Nigeria	Case	NT	NT	488	ST10 Cplx
D05	1995	Nigeria	Case	NT	NT	489	None
G110b	1995	Nigeria	Control	NT	NT	491	None
E100869	1994	UK	Case	125ab	27	495	None
E105835	1994	UK	Case	125ab	H?	495	None
C83	1995	Nigeria	Control	NT	NT	496	None

G125	1995	Nigeria	Control	NT	NT	499	None
G150a	1995	Nigeria	Control	NT	NT	499	None
G112	1995	Nigeria	Control	NT	NT	500	None
G144a	1995	Nigeria	Control	NT	NT	501	ST501 Cplx
E092830	1993	UK	Case	86	11	501	ST501 Cplx
E097500	1994	UK	Case	73	1	501	ST501 Cplx
G122	1995	Nigeria	Control	NT	NT	502	None
93880675	2009	UK	Case	O?	H?	504	None
G83	1995	Nigeria	Case	NT	NT	506	None
C04	1995	Nigeria	Case	NT	NT	507	ST501 Cplx
G155	1995	Nigeria	Control	NT	NT	510	ST10 Cplx
G112a	1995	Nigeria	Control	NT	NT	511	None
C16	1995	Nigeria	Case	NT	NT	512	ST31 Cplx
G115	1995	Nigeria	Control	NT	NT	513	None
G67	1995	Nigeria	Case	NT	NT	515	None
G155a	1995	Nigeria	Control	NT	NT	520	ST10 Cplx
G113	1994	Nigeria	Control	NT	NT	556	ST10 Cplx
G01b	1995	Nigeria	Case	NT	NT	557	None
900770	2007-2011	Bangladesh	Case	69	4	678	ST678 Cplx
601155	2007-2011	Bangladesh	Control	O?	30	678	ST678 Cplx
23280788	2012	UK	Case	104	4	678	ST678 Cplx
25280573	2012	UK	Case	104	4	678	ST678 Cplx
31920216	2013	UK	Case	104	4	678	ST678 Cplx
31920218	2013	UK	Case	104	4	678	ST678 Cplx
31941062	2013	UK	Case	104	4	678	ST678 Cplx
31941063	2013	UK	Case	104	4	678	ST678 Cplx
31941070	2013	UK	Case	104	4	678	ST678 Cplx
12180280	2011	UK	Case	104	4	678	ST678 Cplx
900693	2007-2011	Bangladesh	Case	44	H-	720	ST720 Cplx
900063	2007-2011	Bangladesh	Case	O?	23	720	ST720 Cplx
601175	2007-2011	Bangladesh	Case	8	9	720	ST720 Cplx
601176	2007-2011	Bangladesh	Case	O?	H-	720	ST720 Cplx
900883	2007-2011	Bangladesh	Case	44	23	720	ST720 Cplx
E04	1995	Nigeria	Case	NT	NT	728	None
E099976	1994	UK	Case	113	H-	746	ST746 Cplx
E107252	1994	UK	Control	O?	H?	746	ST746 Cplx
31920214	2013	UK	Case	O?	19	746	ST746 Cplx
900020	2007-20112007-2011	Bangladesh	Case	O?	7	841	ST295 Cplx
31320944	2013	UK	Case	65	H?	841	ST295 Cplx
31740867	2013	UK	Case	65	8	841	ST295 Cplx
E107532	1994	UK	Case	O?	H?	937	None
G80a	1995	Nigeria	Case	NT	NT	940	None
E100867	1994	UK	Case	O?	H?	1114	ST165 Cplx
E98527	1994	UK	Case	19	H-	1114	ST165 Cplx
767	2008	UK	Case	59	H-	1136	None

7092	2007-2011	Bangladesh	Control	125ac	8	1295	None
900108	2007-2011	Bangladesh	Case	21	8	1295	None
900550	2007-2011	Bangladesh	Case	128	12	1326	None
E093414	1993	UK	Control	128ac	H?	1326	None
642	2008	UK	Case	68	17	1380	ST394 Cplx
E107757	1995	UK	Control	O?	H?	1380	ST394 Cplx
E105839	1994	UK	Control	O?	H?	1380	ST394 Cplx
E107247	1994	UK	Case	O?	H?	1380	ST394 Cplx
E108829	1995	UK	Case	O?	H?	1380	ST394 Cplx
E96386	1994	UK	Case	73	18	1380	ST394 Cplx
22980178	2012	UK	Case	O?	18	1380	ST394 Cplx
1627	2008	UK	Case	68	18	1380	ST394 Cplx
1037	2007-2011	Bangladesh	Case	159	23	1490	None
E89099	1993	Bangladesh	Case	28ab	18	1657	None
31941065	2013	UK	Case	63	12	1664	ST295 Cplx
900732	2007-2011	Bangladesh	Case	O?	7	1891	ST1891 Cplx
900769	2007-2011	Bangladesh	Case	69	4	1891	ST1891 Cplx
600965	2007-2011	Bangladesh	Case	126	7	1891	None
601174	2007-2011	Bangladesh	Control	78	7	1891	ST1891 Cplx
E89106	1993	Bangladesh	Case	R	7	1891	ST1891 Cplx
7004	2007-2011	Bangladesh	Control	O?	26	2067	None
7067	2007-2011	Bangladesh	Control	86	27	2166	None
601106	2007-2011	Bangladesh	Control	O?	23	2186	None
601180	2007-2011	Bangladesh	Control	O?	23	2186	None
E101098	1994	UK	Case	O?	H?	2517	ST295 Cplx
601087	2007-2011	Bangladesh	Case	O?	45	3051	None
600961	2007-2011	Bangladesh	Case	69	38	3107	None
E110912	1995	UK	Case	86	27	3570	ST295 Cplx
2866	2008	UK	Case	55	4	3570	ST295 Cplx
7071	2007-2011	Bangladesh	Case	130	35	3670	None
601158	2007-2011	Bangladesh	Case	2	H-	3738	None
900545	2007-2011	Bangladesh	Case	181	16	3748	ST295 Cplx
601110	2007-2011	Bangladesh	Case	25	7	3748	ST295 Cplx
7089b	2007-2011	Bangladesh	Control	O?	27	3748	ST295 Cplx
7089	2007-2011	Bangladesh	Control	181	16	3748	ST295 Cplx
8120	2007-2011	Bangladesh	Case	R	16	3748	ST295 Cplx
E111261	1995	UK	Case	O?	H?	3748	ST295 Cplx
1038	2007-2011	Bangladesh	Case	25	H-	3931	None
3036	2007-2011	Bangladesh	Control	113	H-	DLV	None
7121	2007-2011	Bangladesh	Control	77	18	DLV	None
900553	2007-2011	Bangladesh	Case	51	12	DLV	None
900114	2007-2011	Bangladesh	Case	127	11	DLV	ST40 Cplx
600982	2007-2011	Bangladesh	Control	166	15	DLV	None
7028	2007-2011	Bangladesh	Control	44	34	DLV	ST31 Cplx
E107756	1995	UK	Case	131	H-	DLV	ST10 Cplx
E111268	1995	UK	Case	O?	H?	DLV	ST10 Cplx

1773	2008	UK	Case	O?	30	DLV	None
1027	2007-2011	Bangladesh	Case	O?	H-	SLV	ST10 Cplx
900616	2007-2011	Bangladesh	Case	113	H-	SLV	ST10 Cplx
900654	2007-2011	Bangladesh	Case	O?	18	SLV	ST38 Cplx
900851	2007-2011	Bangladesh	Case	125ac	11	SLV	None
900794	2007-2011	Bangladesh	Case	O?	10	SLV	None
900753	2007-2011	Bangladesh	Case	166	15	SLV	None
900575	2007-2011	Bangladesh	Case	O?	10	SLV	ST10 Cplx
600974	2007-2011	Bangladesh	Case	15	34	SLV	ST31 Cplx
600955	2007-2011	Bangladesh	control	175	1	SLV	ST40 Cplx
600985	2007-2011	Bangladesh	Case	77	34	SLV	ST155 Cplx
601010	2007-2011	Bangladesh	Case	15	23	SLV	ST31 Cplx
601017	2007-2011	Bangladesh	Control	175	28	SLV	ST40 Cplx
601035	2007-2011	Bangladesh	Control	117	27	SLV	ST10 Cplx
601062	2007-2011	Bangladesh	Case	2	42	SLV	None
601068	2007-2011	Bangladesh	Case	2	42	SLV	ST40 Cplx
601098	2007-2011	Bangladesh	Control	O?	45	SLV	None
601101	2007-2011	Bangladesh	Case	O?	23	SLV	None
601173	2007-2011	Bangladesh	Control	O175	31	SLV	ST40 Cplx
601221	2007-2011	Bangladesh	Control	84	27	SLV	ST295 Cplx
601226	2007-2011	Bangladesh	Case	O?	27	SLV	ST295 Cplx
900500	2007-2011	Bangladesh	Case	128ab	12	SLV	ST165 Cplx
E89095	1993	Bangladesh	Case	80	27	SLV	ST155 Cplx
E89112	1993	Bangladesh	Case	69	11	SLV	ST295 Cplx
7079	2007-2011	Bangladesh	Case	25	H-	SLV	ST295 Cplx
7142	2007-2011	Bangladesh	Control	89	H-	SLV	ST10 Cplx
7116	2007-2011	Bangladesh	Control	O?	36	SLV	ST10 Cplx
7155	2007-2011	Bangladesh	Case	25	H-	SLV	None
7201	2007-2011	Bangladesh	Control	58	51	SLV	ST10 Cplx
8016	2007-2011	Bangladesh	Case	O?	10	SLV	ST10 Cplx
8095	2007-2011	Bangladesh	Control	86	30	SLV	ST38 Cplx
8130	2007-2011	Bangladesh	Control	O?	34	SLV	ST38 Cplx
3060197	2010	UK	Case	O?	H?	SLV	None
E099973	1994	UK	Case	151	11	SLV	ST10 Cplx
E099978	1994	UK	Case	80	8	SLV	ST165 Cplx
E103621	1994	UK	Case	73	8	SLV	ST30 Cplx
E104975	1994	UK	Case	O?	H?	SLV	ST31 Cplx
E105358	1994	UK	Control	O?	H?	SLV	None
E107250	1994	UK	Case	O?	H?	SLV	ST746 Cplx
E107530	1994	UK	Case	O?	H?	SLV	ST10 Cplx
E094704	1993	UK	Case	R	H?	SLV	ST10 Cplx
E094706	1994	UK	Case	111ab	H-	SLV	ST40 Cplx
E096617	1994	UK	Case	R	1	SLV	ST86 Cplx
E097480	1994	UK	Case	6	10	SLV	ST10 Cplx
E55280	1988	UK	Case	126	27	SLV	ST40 Cplx
E57144	1989	UK	Case	111	21	SLV	ST40 Cplx

E58583	1989	UK	Case	77	18	SLV	ST31 Cplx
E72376	1991	UK	Case	33	16	SLV	ST295 Cplx
E96390	1994	UK	Case	O?	H-	SLV	ST10 Cplx
1360102	2010	UK	Case	O?	H?	SLV	ST10 Cplx
4220291	2010	UK	Case	O?	10	SLV	ST10 Cplx
4400276	2010	UK	Case	O?	H-	SLV	ST746 Cplx
13160257	2011	UK	Case	O?	H-	SLV	ST746 Cplx
22780065	2012	UK	Case	O?	H?	SLV	ST10 Cplx
23340465	2012	UK	Case	O?		SLV	ST13 Cplx
23980248	2012	UK	Case	181	H?	SLV	ST746 Cplx
2700	2008	UK	Case	O?	10	SLV	ST10 Cplx
900442	2007-2011	Bangladesh	Case	121	H?	SLV	None
3026	2007-2011	Bangladesh	Control	O?	27	TLV	3LV
900245	2007-2011	Bangladesh	Case	161	H-	TLV	None
